# Computational insights into a protease inhibitor from *Streptomyces globosus* VITSMAB-2 molecular docking and dynamics simulations against SARS-CoV-2 main protease

**DOI:** 10.1038/s41598-025-31329-y

**Published:** 2025-12-13

**Authors:** Shatakshi Mishra, Stany Bala Kumar, Aparana Kumari, K. V. Bhaskara Rao

**Affiliations:** 1 School of Bio Sciences and Technology, Vellore Institute of Technology, Vellore, 632014 Tamil Nadu India; 2 Marine Biotechnology Laboratory, Department of Biomedical Sciences, School of Bio Sciences and Technology, Vellore Institute of Technology, Vellore, 632014 Tamil Nadu India

**Keywords:** *Streptomyces globosus* VITSMAB-2, Cysteine protease inhibitor, Phenyl carbamate, ADMET, Molecular docking, SARS-CoV-2 main protease., Biochemistry, Chemical biology, Chemistry, Computational biology and bioinformatics, Drug discovery, Microbiology

## Abstract

Viral proteases are critical components in the life cycles of many dangerous viruses, playing a direct role in facilitating viral replication. Targeting these enzymes through inhibition offers a promising strategy for advancing antiviral agents. This study explores the potential of pigmented actinomycetes from high-altitude terrestrial environments as sources of antiviral agents against SARS-CoV-2, with a particular focus on identifying protease inhibitors. From this unique ecological niche, *Streptomyces globosus* VITSMAB2 was isolated and identified as a promising candidate due to its significant protease-inhibiting capabilities. Both qualitative and quantitative assays confirmed its strong inhibitory activity against key proteases, especially cysteine and serine proteases such as papain and trypsin. Protease inhibitory compounds were partially purified using Ultra-Performance Liquid Chromatography (UPLC), and their identities were determined through Gas Chromatography–Mass Spectrometry (GC-MS) analysis. Among the identified compounds, phenyl carbamate was the most prevalent and emerged as the lead molecule as protease inhibitor. Molecular docking studies revealed that phenyl carbamate exhibited strong binding interactions with the main protease (M-pro) of SARS-CoV-2, highlighting its potential as an antiviral agent. Additionally, assessments of the compound’s drug-likeness and ADME/T (absorption, distribution, metabolism, excretion, and toxicity) profiles indicated favorable pharmacokinetic properties, supporting its candidacy for therapeutic development. Molecular dynamics simulations further confirmed the stability of the phenyl carbamate-M-protease complex, reinforcing the compound’s antiviral potential. Therefore, phenyl carbamate shows considerable promise as a lead antiviral compound and merits further validation through extensive in vitro and in vivo experimentation to fully assess its therapeutic efficacy.

**Supplementary Information:**

The online version contains supplementary material available at 10.1038/s41598-025-31329-y.

## Introduction

Coronaviruses (CoV) comprise a large viral family, recognized for their crown-like shape, which primarily cause respiratory illnesses. They range from causing the common cold to severe illnesses like Middle East Respiratory Syndrome (MERS) and severe acute respiratory syndrome (SARS). A novel coronavirus (nCoV) appeared in 2019, previously unknown in humans. CoV are zoonotic, meaning they typically originate in animals and can transfer to humans, as seen with SARS-CoV from civet cats and MERS-CoV from dromedary camels. While many animal CoV do not affect humans, some can cross over and spread between humans, as with the CoV causing COVID-19^1^.

The COVID-19 pandemic, caused by SARS-CoV-2, marks the third major coronavirus outbreak of the 21st century, following SARS-CoV and MERS-CoV. These viruses, which belong to the β coronavirus genus, share a high sequence identity, particularly in their main protease (Mpro). Mpro, a 306-residue protease featuring a cysteine-histidine catalytic dyad, is critical for viral polyprotein processing and thus represents a prime therapeutic target due to its essential role in viral replication and maturation. Mpro cleaves the viral polyproteins pp1a and pp1ab at specific sites, producing functional viral proteins necessary for viral replication within host cells. This protease’s activity hinges on its catalytic dyad, composed of cysteine (Cys 145) and histidine (His 41), classifying it as a cysteine protease. Mpro is nearly inactive in its monomeric form and requires dimerization for full enzymatic activity. This dimerization occurs through interactions between the N-terminus of one monomer and domain II of another, forming a fully functional enzyme capable of processing viral polyproteins. Targeting Mpro is a promising strategy for antiviral therapy because inhibiting this protease can effectively halt viral replication. Inhibitors designed to target Mpro can bind to its active site or other crucial regions, preventing the protease from processing the viral polyproteins. This disruption results in the production of non-functional viral proteins, thereby stopping the virus from replicating and spreading. The well-characterized structure of Mpro facilitates the rational design of specific inhibitors. High-throughput screening and structure-based drug design have identified several promising Mpro inhibitors that demonstrate strong antiviral activity and low cytotoxicity. These inhibitors often mimic the natural substrates of Mpro, binding to the active site and blocking its enzymatic activity. Targeting Mpro offers a direct and effective approach to developing antiviral therapies against SARS-CoV-2, disrupting the virus’s life cycle and reducing its pathogenicity^2,3^.

One promising source of viral protease inhibitors (PIs) is Actinomycetes, microorganisms found in diverse geographical niches, ranging from terrestrial to marine environments. Actinomycetes are well-documented producers of antiviral compounds, as reported by various research groups. A study conducted by Kamarudheen et al. focused on the extraction and characterization of a PI from *Streptomyces griseoincarnatus* HK12, demonstrating antiviral activity against the Chikungunya virus (CHIKV). The PI exhibited inhibitory effects against papain and trypsin proteases and the high molecular weight PI (66–70 kDa) significantly suppressed cytopathic effects in Baby Hamster Kidney Fibroblast (BHK21) cells with an EC_50_ of 11.21 µg/mL, marking it as the first natural PI against CHIKV^4^. Angelova et al. purified and characterized a novel protease inhibitor, PISC-2002, from Streptomyces chromofuscus culture supernatants. It demonstrated broad inhibition of serine proteases and notable antiviral activity against influenza A/Rostock/34 (H7N7)^5^.

**Fig. 1:**
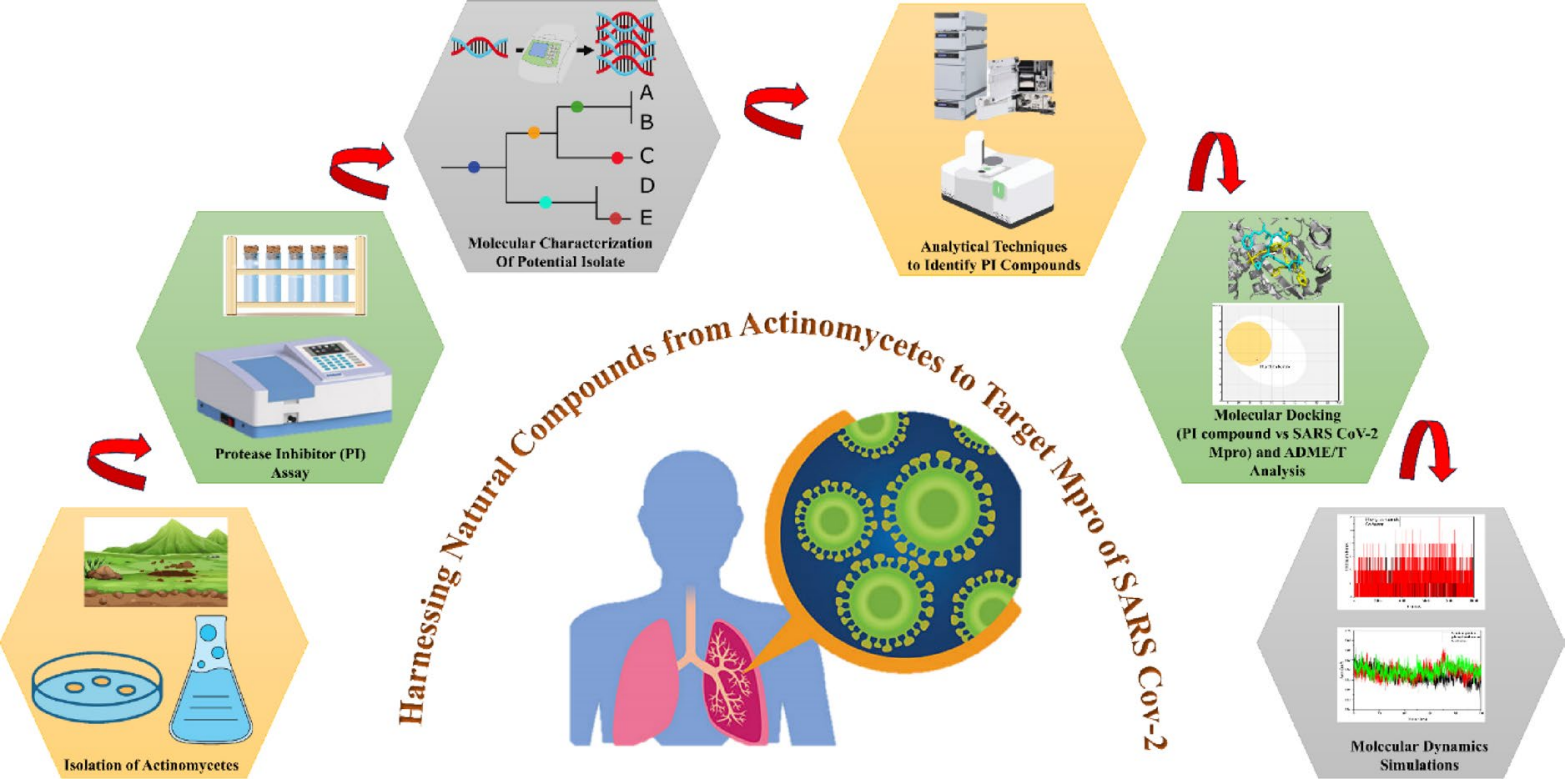
Workflow depicting the isolation of actinomycetes, PI screening and characterization, analytical identification, in silico docking with ADME/T evaluation, and molecular dynamics simulations targeting SARS-CoV-2 Mpro.

The present research provides new perspectives on the possible interactions between a cysteine PI derived from terrestrial actinomycetes and the SARS-CoV-2 Mpro receptor, a critical component in coronavirus pathogenesis, through in silico analysis (Fig. 1). The study began with the isolation and characterization of PI-producing higher-altitude terrestrial actinomycetes. The PI compound was identified through qualitative and quantitative protease inhibition assays, partially purified, and subsequently analyzed for its therapeutic potential. Molecular docking analysis was conducted to evaluate the compatibility of the inhibitor with the SARS-CoV-2 Mpro receptor. Key parameters such as docking scores and binding energies were assessed, with a primary focus on determining binding energy as a measure of interaction strength and stability. Benchmarking against existing inhibitory cofactors ensured the accuracy and reliability of the findings. Following docking studies, drug-likeness and ADMET (absorption, distribution, metabolism, excretion, and toxicity) analyses were performed. These evaluations revealed favorable pharmacokinetic properties, supporting the inhibitor’s potential as a therapeutic agent. This approach quickly screens candidate compounds, accelerating drug discovery with high accuracy and precision, and ultimately supports the development of new SARS-CoV-2 therapies through computational analysis.

Considering the latest advancements, we propose employing computational approaches as an alternative to traditional wet lab methods for studying viruses. This approach offers several advantages, including saving time, reducing costs, and minimizing the risk of contamination. By harnessing computational tools, we can swiftly explore potential interactions between a cysteine PI from terrestrial actinomycetes and the SARS-CoV-2 Mpro receptor, crucial in coronavirus pathogenesis. This innovative strategy allows for rapid screening and evaluation of candidate compounds, expediting the drug discovery process while maintaining high accuracy and reliability. Ultimately, this research paves the approach to development of novel therapies and cures against SARS-CoV-2, capitalizing on the efficiency and precision offered by computational analysis.

## Materials and methods

### Sample collection

The soil sample was collected in December 2023 from Eravikulam National Park, Munnar, Kerala, India, under strict aseptic conditions. To maintain sample integrity and prevent contamination, the sample was carefully transferred into a sterile Ziplock bag immediately upon collection. These precautionary measures were implemented to preserve the native microbial diversity characteristic of the high-altitude ecosystem of the Western Ghats, ensuring the reliability and accuracy of subsequent analyses.

### Isolation of pigmented actinomycetes

To isolate actinomycetes from the collected soil sample, Actinomycetes Isolation Agar (AIA) and Starch Casein Agar (SCA) were utilized as selective media. The preliminary screening was conducted using the spread plate method following the preparation of serial dilutions of the soil suspension, ranging from 10⁻¹ to 10⁻⁶. Individual colonies were subsequently isolated and purified through repeated subculturing on the same media. The inoculated plates were incubated at 25 °C for a period of seven days to promote the growth and development of distinct actinomycete colonies^6–8^.

### Identification and characterization of pure isolates

After obtaining pure cultures, each isolate was carefully analyzed for morphological characterization. Key features such as colony colour, shape, surface texture, pigment production, and margin type were observed and systematically recorded under suitable lighting conditions. This detailed morphological assessment supported accurate classification and differentiation of the actinomycete strains, laying a reliable foundation for further research. Additionally, the isolates were cultured on ISP media 1 through 7 and Nutrient Agar (NA), following the standardized protocols outlined by the International Streptomyces Project to facilitate comprehensive characterization^4,8^.

### Production of secondary metabolites

Starch Casein Broth was formulated and inoculated with all pigmented actinomycete isolates to promote growth and pigment synthesis. The cultures were incubated at 28 °C with constant agitation at 100 rpm for seven days to ensure optimal metabolic activity. Post-incubation, the broths were visually inspected for notable pigmentation and other distinguishable characteristics. To isolate the cellular and extracellular components, the cultures were subjected to ultracentrifugation followed by filtration. The resulting supernatants and cell pellets were then stored under refrigerated conditions to preserve their consistency for subsequent protease inhibitor (PI) activity evaluation^4,8,9^.

### Protease inhibitor assays (qualitative and quantitative)

#### Qualitative assay of papain and trypsin inhibitor

Casein Agar medium was prepared, autoclaved prior to use. Sterile wells were carefully made on the surface of the solidified agar under aseptic conditions. Each well was loaded with one of the following: distilled water (serving as the control), the crude protease inhibitor (PI) extract, and papain (1 mg/mL) (a standard cysteine protease). The plates were incubated for 24 h and then examined for inhibition zones. An identical protocol was applied using trypsin, a standard serine protease, to assess inhibition activity^4,8^.

#### Quantitative assay of papain and trypsin inhibitor

Under aseptic conditions, 150 µL of the crude extract was combined with an equal volume of papain solution prepared in Tris-HCl and CaCl₂ buffer at pH 7.5. Subsequently, 210 µL of Tris-HCl buffer (pH 7.4) was added, and the contents were gently vortexed and incubated at 37 °C for 15 min to enable interaction. After this, 310 µL of casein solution, prepared in Tris-HCl buffer at pH 8.0, was added to the mixture, which was then incubated again at 37 °C to promote proteolytic activity. The reaction was halted by the addition of seventy microlitres of glacial acetic acid. Absorbance was measured at 410 nm to evaluate enzyme inhibition. The same protocol was followed using trypsin in place of papain to assess serine protease inhibition^8,9^. The percentage of protease inhibition exhibited by each isolate was compared to identify the isolate demonstrating the highest activity.

### Molecular identification and characterization of the potential isolate

Genomic DNA was extracted from the selected actinomycete isolate employing the DNA Mini Kit (QIAGEN, Hilden, Germany). The procedure included emulsifying the bacterial colonies in saline, followed by enzymatic treatments with lysozyme and proteinase K. Lysis was carried out using AL buffer and ethanol, and DNA was subsequently purified using spin-column chromatography. For molecular identification, the 16 S rRNA gene was amplified via polymerase chain reaction (PCR) using universal eubacterial primers 27 F (5′AGAGTTTGATCCTGGCTCAG-3′) and 1492R (5′TACGGYTACCTTGTTACGACTT-3′) in a Veriti 96-well Thermal Cycler. The resulting PCR products were assessed through agarose gel electrophoresis, purified, and sequenced using the ABI 3730XL DNA sequencer. The obtained sequence data were refined and compared against NCBI reference sequences for taxonomic identification. Phylogenetic analysis of the 16 S rRNA gene sequence was conducted using MEGA-X version 10.1.8. Similar sequences were identified through NCBI’s Nucleotide BLAST tool. Multiple sequence alignment was performed using the ClustalW algorithm with default gap penalties. A phylogenetic tree was constructed using the maximum likelihood approach, and evolutionary distances were calculated using the Jukes-Cantor model. The reliability of the tree was assessed with 500 bootstrap replications^10^.

### Morphological and microscopic characterization of the potential isolate

To examine the spore chain morphology of the potential isolate, the organism was cultured on Starch Casein Agar (SCA) with sterile coverslips and incubated for 48 h. Following incubation, the coverslips were subjected to critical point drying to preserve structural integrity and subsequently mounted onto aluminium stubs using conductive adhesive. Scanning Electron Microscopy (SEM) was then employed to visualize the detailed cellular and spore chain morphology. Additionally, microscopic identification was supported by Gram staining to determine the cellular characteristics of the isolate^8^.

### Identification of PI compound: organic or aqueous phase in solvent-solvent extraction

The crude extract (as described in “Production of secondary metabolites”) was mixed with an equal volume of ethyl acetate and carefully poured into a separating funnel, ensuring minimal formation of air bubbles during the transfer. The mixture was left undisturbed at room temperature for 24 h to allow complete phase separation. After this period, the organic and aqueous layers were clearly separated. Based on qualitative results showing the largest inhibition zone and quantitative data indicating the highest inhibition percentage, papain was chosen as the standard cysteine protease for subsequent analyses.

#### Quantitative assay to determine the activity at the organic phase

Under sterile conditions, 150 µL of the crude extract was mixed with an equal volume of papain solution prepared in Tris-HCl and CaCl₂ buffer at pH 7.5. This was followed by the addition of 210 µL of Tris-HCl buffer with a pH of 7.4. The components were thoroughly mixed and incubated at 37 °C for 15 min to allow interaction. Thereafter, 310 µL of casein solution, prepared in Tris-HCl buffer at pH 8, was added, and the mixture was further incubated at 37 °C. To halt the enzymatic reaction, 70 µL of glacial acetic acid was added. The extent of the reaction was assessed by measuring absorbance at 410 nm. This procedure was applied to both the organic and aqueous phases to evaluate and compare the protease inhibitory activity of each phase of the isolate.

### Characterization of PI compound employing analytical techniques

#### UPLC analysis

The supernatant containing the protease inhibitor from the selected isolate was analyzed using Ultra Performance Liquid Chromatography (UPLC) to evaluate its purity. The analysis was performed using a Waters Acquity H-Class UPLC system equipped with a photodiode array (PDA) detector. For the active fraction, the mobile phase consisted of 70% (v/v) acetonitrile in Milli-Q water, delivered at a constant flow rate of 1 mL/min. The chromatographic run was carried out over a 20-minute period, during which retention times and peak profiles were carefully documented. The partially purified fractions obtained through UPLC were then evaluated through quantitative PI assays. The same purified fraction was subsequently subjected to compound identification using Gas Chromatography–Mass Spectrometry (GC-MS)^11^.

#### GC-MS analysis

Gas Chromatography–Mass Spectrometry was employed to identify the bioactive compounds present in the active UPLC fractions. The analysis employed a fused silica GC column (Elite-5 MS; 5% biphenyl, 95% dimethylpolysiloxane) with dimensions of 30 m × 0.25 mm internal diameter × 250 μm film thickness. The procedure was conducted on a PerkinElmer GC Clarus 680 system (30 m × 0.25 mm × 0.25 μm), coupled with a Clarus 600 EI Mass Spectrometer (PerkinElmer, Waltham, MA, USA). A 1 µL volume of the sample was injected, and the temperature was programmed to rise from 65 °C to 300 °C at a rate of 10 °C per minute, followed by a final isothermal phase lasting 2 min. Mass spectrometry outputs were documented using electron ionization (EI). The resulting data were analyzed using TurboMass software, version 5.4.2, to interpret and identify the chemical constituents within the fractions^11^. The compound with the highest area percentage from the GC-MS chromatogram analysis was shortlisted for further molecular docking analysis.

### Molecular docking analysis of the PI with SARS-CoV-2 m-protease (cysteine protease)

Molecular docking is performed to evaluate the binding affinity of PIs with SARS-Cov-2 Mpro. This process helps confirm the interaction between the compound under study and the target, providing valuable insights into their potential therapeutic applications. The structure of the SARS-CoV-2 Mpro, a cysteine protease, was obtained from the Protein Data Bank (PDB ID: 6Y2F). This structure was co-crystallized with an alpha-ketoamide inhibitor. To confirm the reliability of the binding sites, the identical ligand was re-docked into the protease using AutoDockTools 1.5.7^12^. The resulting re-docked complex was superimposed onto the original structure, demonstrating a high degree of consistency in the binding site locations. Subsequently, the PI compound, identified in this study, was docked against the same protein structure (PDB ID: 6Y2F) at the identified active sites. The docking procedure utilized a grid box center and the dimension coordinates based on the active site residues. Discovery studio and Maestro Schrodinger were used to visualise the 2D interactions between the protein and the inhibitors, respectively^13,14^.

### Drug-likeness, and ADME/T characteristics of the PI compound

The PI identified is screened for its, ADME/T using SwissADME (http://www.swissadme.ch/index.php) (accessed on 18 May 2024)^15^, molsoft (https://molsoft.com/mprop/) (accessed on 18 May 2024), and ADMETlab 2.0 (https://admetmesh.scbdd.com/) (accessed on 18 March 2024)^16^. In silico ADME/T and drug-likeness assessments enhance drug discovery by quickly and cost-effectively predicting pharmacokinetic and toxicity profiles, identifying potential issues early, and guiding the optimization of candidates. The preliminary screening of metabolites was performed using Lipinski’s Rule of Five, a guideline for assessing drug-likeness. This rule evaluates critical parameters, including molecular weight (< 500 g/mol), lipophilicity/logP (< 5), hydrogen bond acceptors (< 10), hydrogen bond donors (< 5), and the number of rotatable bonds (< 10)^17,18^. These tools minimize animal testing, boost success rates, and speed up safe drug development.

### MD simulation of the pure compound with SARS-CoV-2 m-protease

Molecular dynamics (MD) simulation is employed to investigate the stability and behavior of the compound within a dynamic biological environment. This method assesses the flexibility of the compound and its interaction network within the system, supporting its potential as a therapeutic agent and verifying the stability of the compound-target complex^19^. MD simulation serves as an alternative to in vitro and in vivo studies, especially when laboratory facilities for virology studies are unavailable. By relying on computational methods, MD simulation helps save time, reduces dependence on animal testing, and cuts down on associated costs. Molecular dynamics (MD) simulations were performed using GROMACS version 2023.1 to evaluate the stability of the metabolite throughout the simulation. The CHARMM36 force field was applied, and the topology of the metabolite was generated using the CGenFF web server. Solvation was carried out using the SPC (Simple Point Charge) water model within a dodecahedral simulation box containing explicit water molecules. To ensure charge neutrality, 0.15 M NaCl (29 Na⁺ and 27 Cl⁻ ions) was added. Energy minimization was performed using the steepest descent algorithm for 50,000 steps to resolve steric clashes and optimize geometry. The system was then equilibrated under NVT and NPT conditions at 300 K and 1 bar. Molecular dynamics simulations were then carried out for a total of 100 ns, generating 5000 frames using the leap-frog integrator. The resulting trajectories were analyzed to assess structural stability and dynamic behavior through parameters such as root mean square deviation (RMSD), root mean square fluctuation (RMSF), radius of gyration (Rg), and solvent-accessible surface area (SASA)^13,18^.

### Statistical analysis

All experiments were performed in triplicate, and the data were statistically evaluated using both one-way and two-way ANOVA to determine significance. Results are expressed as mean values with standard deviation and illustrated using bar graphs. GraphPad Prism version 6.0 was used for the statistical analyses, with significance assessed based on the calculated p-values.

## Results and discussion

### Sample collection

**Fig. 2:**
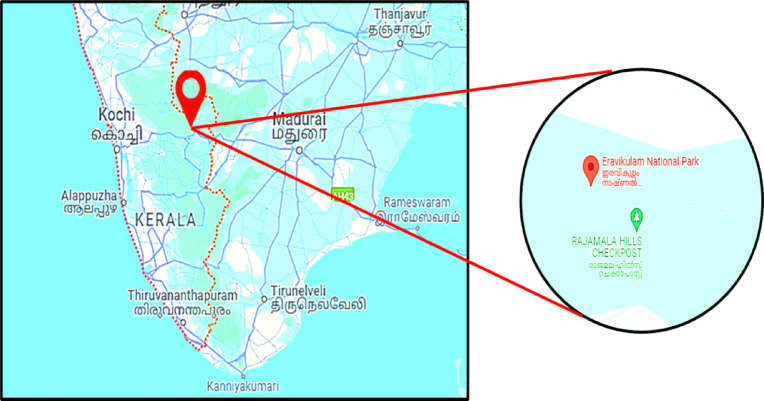
Pictorial representation depicting the geographical location of sample collection from high-altitude terrestrial sources within Eravikulam National Park, Kerala (https://eravikulam.org/).

The sample was aseptically obtained from a specific site located at 10.1454° N latitude and 77.0366° E longitude. Figure 2 provides a pictorial representation of the collection site for visual reference. This step ensures the accurate documentation of the sample’s origin, providing geographical context for reproducibility and ecological relevance in the study.

### Isolation of pigmented actinomycetes

**Fig. 3:**
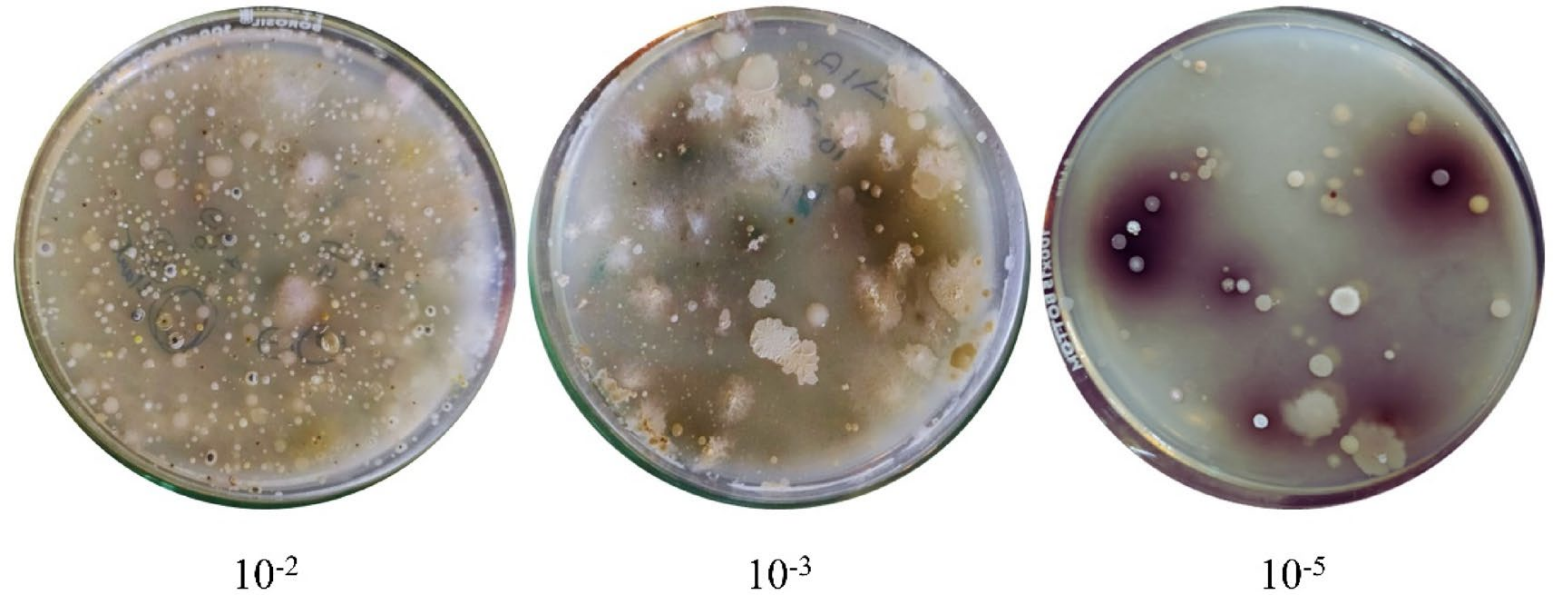
Master plates of terrestrial actinomycetes prepared at various dilutions.

Actinomycetes were isolated from the soil sample using AIA and SCA media through the spread plate method for initial screening. The crowded plate technique was applied, and a high density of actinomycetes was observed at each dilution level. These plates served as the primary or master plates for further analysis. An image for each dilution performed is provided in Fig. 3, showcasing the master plates. This step ensures efficient isolation and preliminary screening of diverse actinomycetes strains, facilitating the identification of potential candidates for bioactive compound production.

### Identification and characterization of pure isolates

All the isolates were subcultured to ensure the production and growth of pure isolates, and are further observed for their morphological characteristics and their ability to form intracellular or extracellular pigment, the same data is tabulated in Table 1. Further, the isolates are checked for their ability to utilize the components present in ISP medium 1–7 and NA, the results are documented in Table 2. The growth patterns of various Streptomyces isolates on different ISP media indicate their nutrient utilization capabilities and metabolic preferences. SMAB2 exhibits significant growth in ISP-1, ISP-2, ISP-5, and ISP-6, suggesting effective utilization of tryptone, soya peptone, yeast extract, malt extract, asparagine, and glycerol, contributing to its white and yellow pigmentation. SMAB6 shows moderate to high growth in ISP-1, ISP-2, ISP-4, ISP-5, and ISP-6, with white and grey colonies indicating reliance on basic nutrients, inorganic salts, and starch. SMAB15 demonstrates high growth in ISP-1, ISP-5, and ISP-6, with distinctive yellow and green colonies, indicating strong use of yeast extract, malt extract, asparagine, and peptone. SMAB16 shows significant growth in ISP-1, ISP-2, ISP-5, and ISP-6, with white and pinkish colonies reflecting the effective use of tryptone, soya peptone, yeast extract, malt extract, and asparagine. SMAB17 and SMAB18 exhibit moderate growth in multiple media types, utilizing inorganic salts, starch, asparagine, and glycerol, with grey and brown pigmentation. SMAB19 shows robust growth in ISP-1, ISP-4, ISP-5, and ISP-6, indicating versatile metabolic capabilities with effective use of tryptone, soya peptone, inorganic salts, starch, asparagine, and glycerol, contributing to its white and grey colonies. These growth patterns highlight the isolates’ metabolic diversity and guide the selection of suitable media for culturing Streptomyces species. All the pigmented isolates are taken further for secondary metabolite production.

**Table 1:** Physical characterization of the pure isolates.

Isolates	Color	Shape	Texture	Pigment production	
				Reverse side	Intracellular
SMAB1	Pale Yellow	Irregular	Powdery and dry	−	−
SMAB2	Silver with White Border	Irregular	Powdery and dry	+ (Brown)	−
SMAB3	Brown with White Border	Irregular	Powdery and dry	−	−
SMAB4	Brown with White Border	Irregular	Powdery and dry	−	−
SMAB5	White	Circular	Cottony	−	−
SMAB6	Brown	Irregular	Dry	+ (Brown)	+ (Brown)
SMAB7	Brown with White Border	Irregular	Powdery and dry	−	−
SMAB8	Yellow	Irregular	Powdery and dry	−	+ (Yellow)
SMAB9	White	Irregular	Powdery and dry	−	−
SMAB10	Silver with White Border	Irregular	Powdery and dry	−	−
SMAB11	Brown with White Border	Irregular	Powdery and dry	−	−
SMAB12	Brown with White Border	Irregular	Powdery and dry	−	−
SMAB13	Pale Yellow	Irregular	Powdery and dry	−	−
SMAB14	White	Circular	Cottony	−	−
SMAB15	Golden Yellow	Circular	Shiny	−	+ (Yellow)
SMAB16	Magenta	Irregular	Shiny	−	+ (Purple)
SMAB17	Grey	Circular	Powdery and dry	+ (Brown)	−
SMAB18	White	Circular	Powdery and dry	+ (Brown)	−
SMAB19	Yellow	Irregular	Powdery and dry	−	+ (Yellow)
SMAB20	Grey	Irregular	Powdery and dry	−	−

**Table 2:** ISP characterization of the selected isolates under study.

Isolates	ISP-1	ISP-2	ISP-3	ISP-4	ISP-5	ISP-6	ISP-7	NA
SMAB2	++ (White)	++ (Yellow)	−	−	++ (Brown with white border)	++ (Yellow)	−	++ (White)
SMAB6	+ (Off White)	++ (Off White)	−	++ (Grey)	+ (White)	++ (white)	−	+ (white)
SMAB15	++ (Yellow)	+ (White)	−	−	++ (Yellow)	+++ (Green colony, black pigment)	−	++ (Pale yellow)
SMAB16	++ (White)	++ (Pinkish)	−	−	+ (Pinkish)	++ (White)	−	−
SMAB17	+ (White)	+ (White)	−	++ (Grey)	++ (Brown)	++ (Yellow)	−	++ (Grey)
SMAB18	+ (Pale yellow)	+ (Grey)	−	++ (Grey)	++ (Brown)	++ (Off White)	−	−
SMAB19	++ (Off White)	+ (Grey)	−	++ (Grey)	++ (Brown)	++ (Off White)	−	++ (white)

### PI assays (qualitative and quantitative)

The Starch Casien broth was successfully changed from colorless to colored broth along with the formation of cell pellets, after 7 days of incubation in a shaker incubator, at 100 rpm.

#### Qualitative assay of papain and trypsin inhibitor

**Fig. 4:**
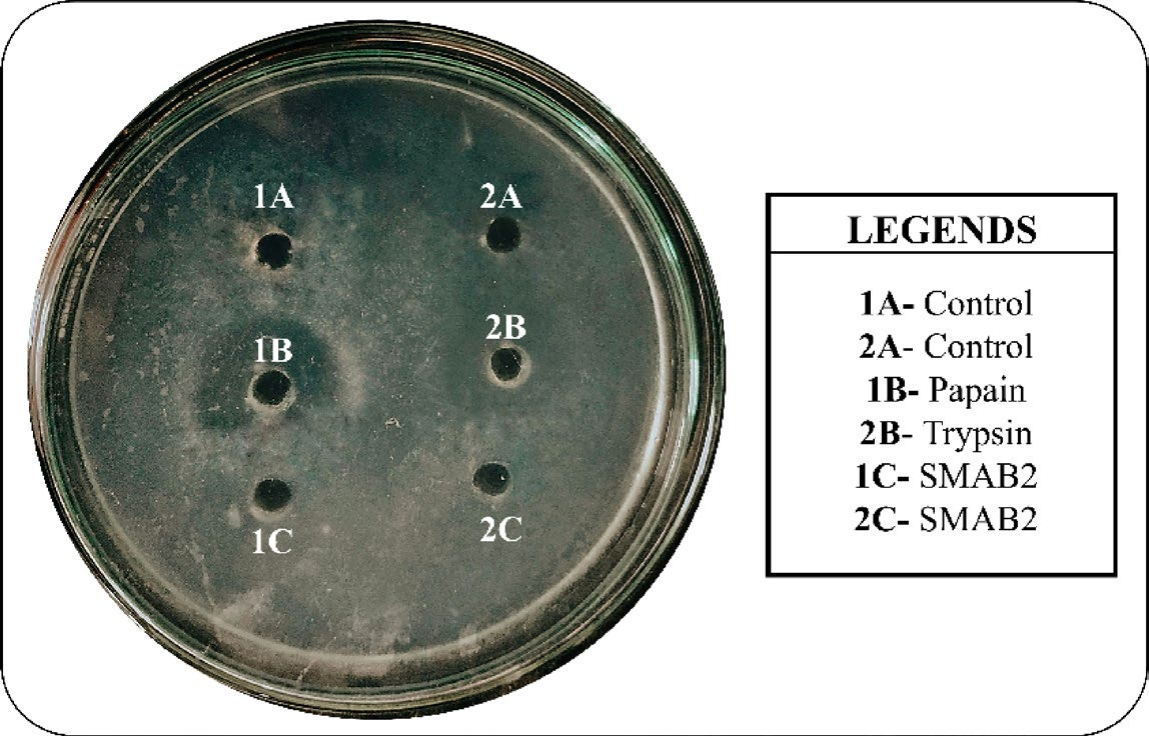
Qualitative plate assay to assess the PI activity of isolate SMAB2.

After 24 h of incubation, clear zone was observed around the papain well (1B), indicating the presence of protease inhibitor (PI) activity. This inhibitory response was exhibited by sample SMAB2 (1 C), as illustrated in Fig. 4. Among the seven isolates tested, only SMAB2 showed a positive result in the qualitative assay for PI activity, establishing it as a promising protease inhibitor. Additionally, the pronounced zone of clearance around the papain well suggests a higher level of inhibition against cysteine proteases compared to serine proteases.

#### Quantitative assay of papain and trypsin inhibitor

**Fig. 5:**
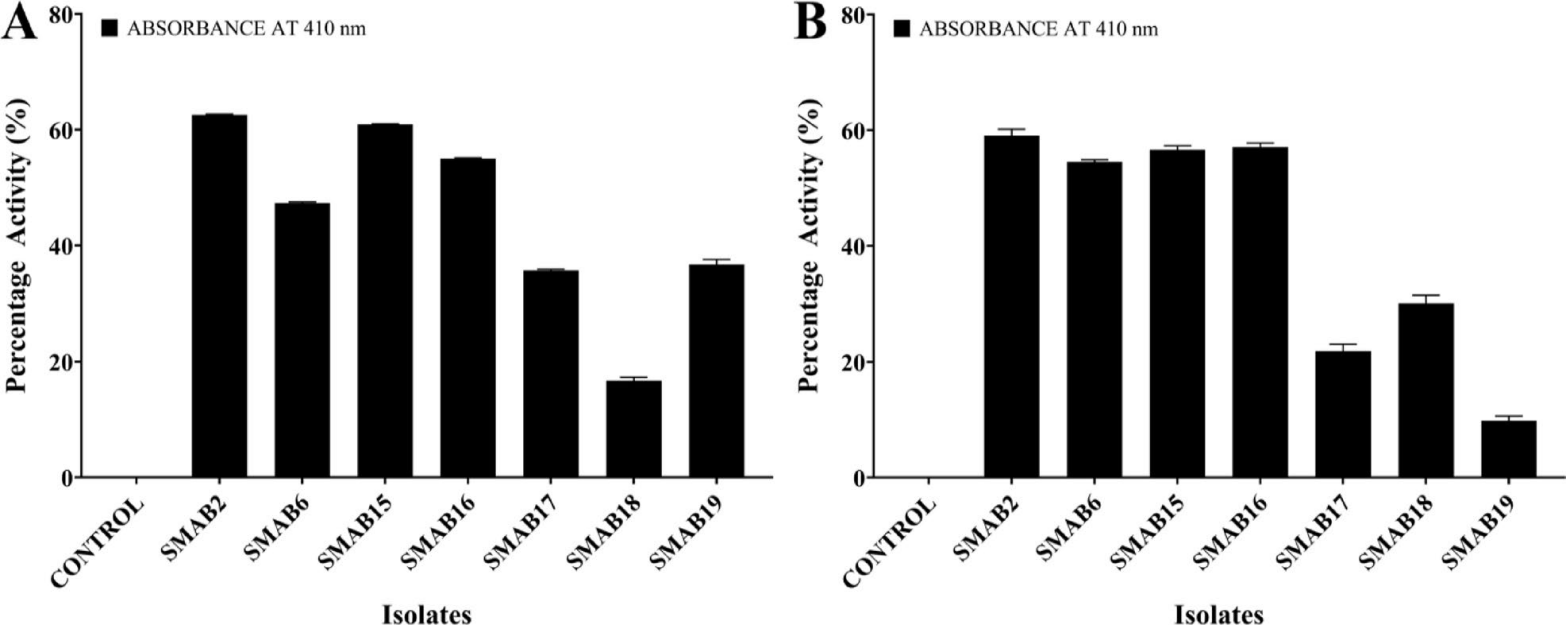
Graphical representation displaying the percentage of protease inhibition activity of each isolate, highlighting their relative efficacy with a significance of *p* < 0.0001. (**A**) cysteine protease (papain) (**B**) Serine protease (trypsin).

Supplementary Tables 1 and 2 provide absorbance measurements at 410 nm for different isolates, comprising the control and several SMAB samples. The data for each isolate include the mean absorbance, standard deviation, and the corresponding percentage of inhibition calculated in comparison to the control. Among the data in Supplementary Table 1, SMAB2 shows minimum recorded absorbance, averaging 0.182 ± 0.003, indicating the highest trypsin inhibition activity among the samples at 59.08%. Conversely, in Supplementary Table 2, SMAB2 exhibits the lowest absorbance with an average of 0.143 ± 0.0005, suggesting the highest papain inhibition activity among the samples at 62.57%. These findings highlight SMAB2’s potential as a potent inhibitor of papain and trypsin activity, indicating its promising candidacy for further investigation. These results accentuate SMAB2’s notable papain inhibition activity compared to other samples analyzed. The graphical representations in Fig. 5A,B complement the presented data, facilitating a comprehensive understanding of the inhibitory activities exhibited by the SMAB isolates against papain and trypsin.

### Molecular identification and characterization of the potential isolate

**Fig. 6:**
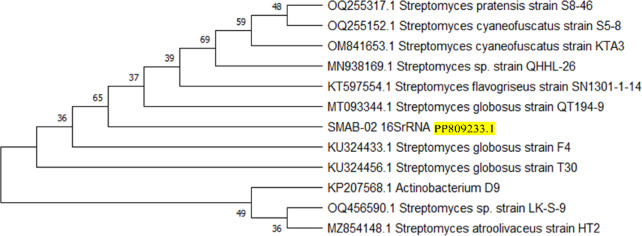
Phylogenetic tree analysis of SMAB2 revealing its evolutionary relationship with other closely related strains.

The data presented in Table 3 details the PCR conditions employed for characterizing the isolate at a molecular level. Supplementary Fig. 1A illustrates a PCR Gel image, and Supplementary Fig. 1B shows the PCR ladder used for size estimation. Together, these confirm an approximate size of 924 base pairs for the isolate, identified as GenBank ID: PP809233.1 *Streptomyces globosus* VITSMAB2. Furthermore, Fig. 6 displays a phylogenetic tree constructed using ClustalW for sequence alignment and the maximum likelihood approach, supported by 500 bootstrap replicates to assess the genetic relatedness between *Streptomyces globosus* VITSMAB2 and related species.

**Table 3:** Optimum conditions to run PCR of SMAB2.

Thermal cycling steps			
Initial denaturation	5 min	96 °C	1 Cycle
Denaturation	40 s	95 °C	32 Cycles
Annealing	25 s	54 °C	
Extension	1 min	70 °C	
Final extension	10 min	70 °C	1 Cycle
Hold	–	4 °C	

### Morphological and microscopic characterization of the potential isolate

**Fig. 7:**
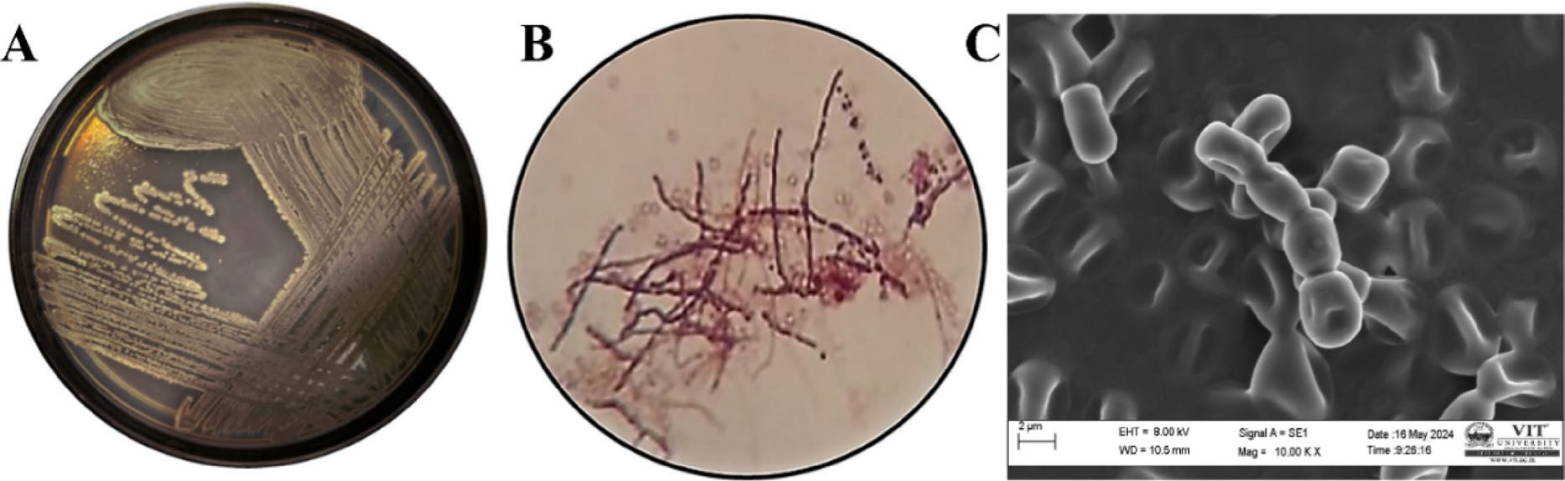
Morphological and microscopic characterization of *Streptomyces Globusus* VITSMAB2. (**A**) Quadrant streaking was performed on SCA (**B**) Gram staining at 100× (**C**) SEM Image at 10,000× magnification.

*Streptomyces Globusus* VITSMAB2 was cultured on SCA, resulting in the observation of greyish-white colonies with brown pigmentation on the reverse side (Fig. 7A), Gram staining (Fig. 7B), visualized under 100x magnification, depicted filamentous rods in a chain formation, appearing purple. Furthermore, SEM analysis at the same magnification revealed a morphology characterized by large beads arranged in chains (Fig. 7C). These findings are crucial for accurate species identification and understanding of the isolate’s biological features, supporting its potential for further study and biotechnological applications.

### Identification of PI compound: organic or aqueous phase in solvent-solvent extraction

#### Quantitative assay to determine the activity at the organic phase

**Fig. 8:**
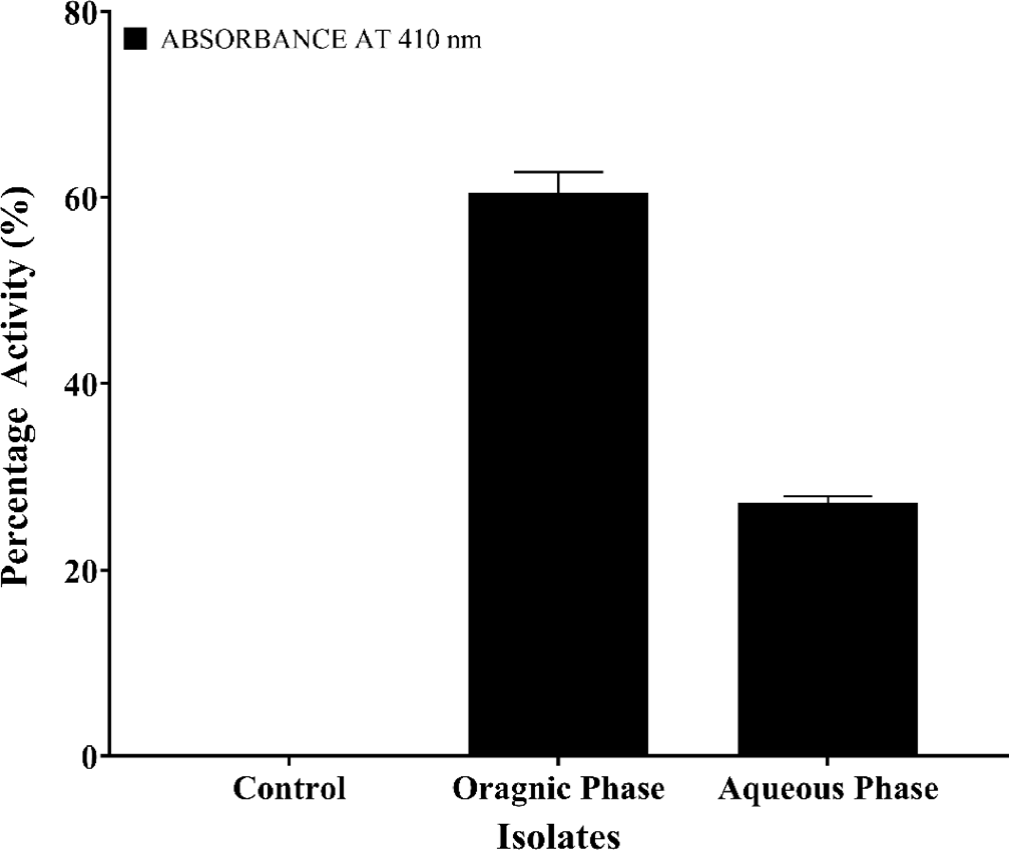
Quantitative protease inhibition assay showing percentage inhibition in both the organic and aqueous phases with a significance of *p* < 0.0001.

A 1:1 ratio of ethyl acetate and crude extract was carefully introduced into a separating funnel. After allowing the mixture to stand undisturbed for 24 h, two distinct layers—aqueous and organic—were formed. The organic layer demonstrated notable protease inhibitory activity, as shown in Supplementary Table 3, which showed absorbance readings at 410 nm for the control and both organic and aqueous phases of SMAB2. The organic phase of SMAB2 exhibited a lower absorbance value (mean: 0.150 ± 0.008) and higher inhibition percentage (60.29%) in comparison to the aqueous phase (mean: 0.277 ± 0.004, 27.29% inhibition). These findings highlight the enhanced inhibitory potential of the organic extract, emphasizing the significant impact of solvent phase on SMAB 2’s bioactivity. A visual comparison is provided in Fig. 8.

### Characterization of PI compound employing analytical techniques

#### UPLC analysis

**Fig. 9:**
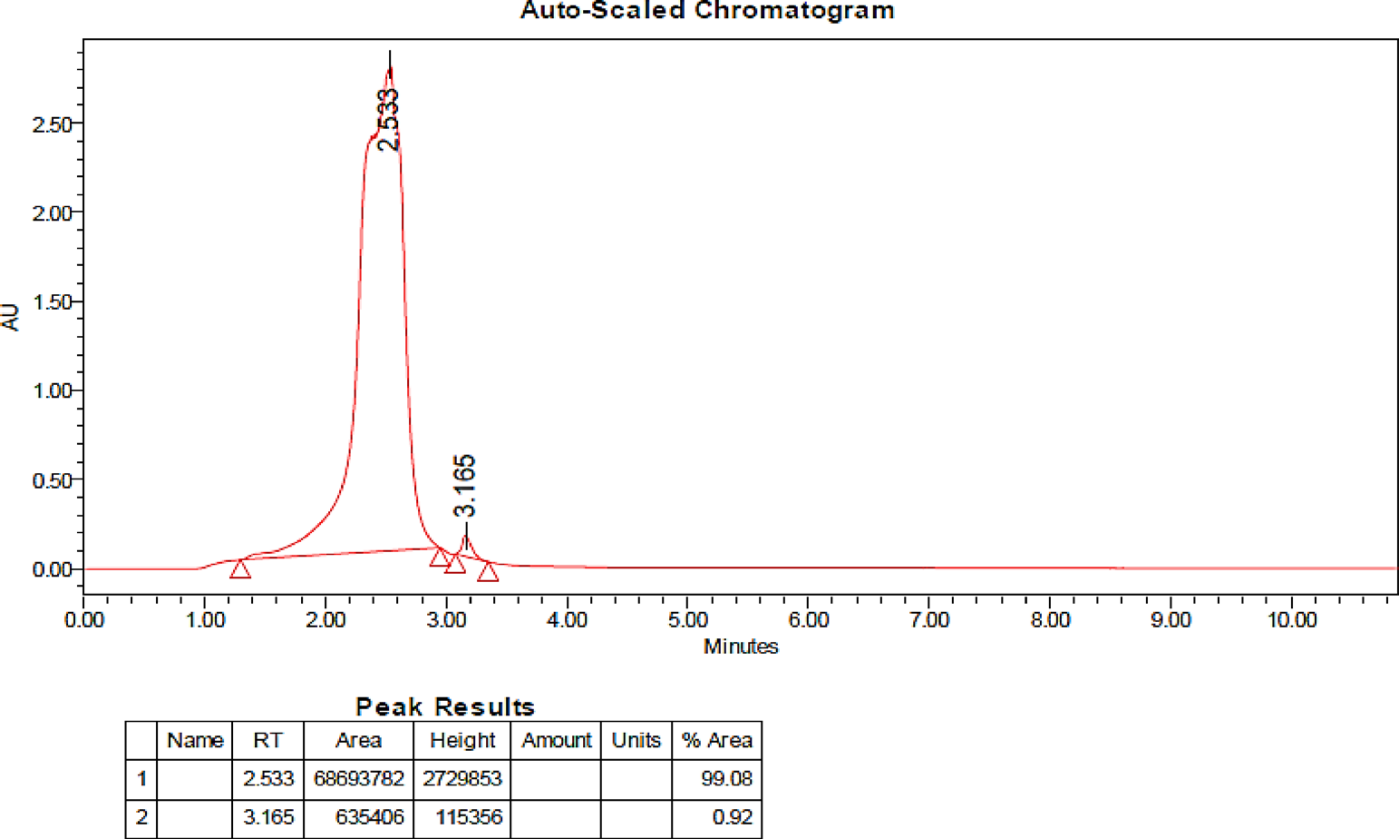
Chromatographic profile generated from UPLC analysis.

The significance of UPLC analysis lies in its ability to separate and identify compounds based on their retention times, providing a high-resolution profile of the sample. The distinct peaks observed at specific retention times allow for the isolation of pure fractions, which can then be subjected to further analysis. Figure 9 displays the chromatographic profile obtained from a 20-minute gradient elution using a mobile phase consisting of acetonitrile and MilliQ water. Two distinct peaks were observed, with the initial peak emerging at 2.533 min and a second peak at 3.533 min. Remarkably, the first peak accounted for 99.08% of the total area, indicating it as the predominant component in the sample. The eluted fraction obtained at a retention time of 2.533 was subjected to quantitative PI activity analysis, performed in triplicates (refer to “Quantitative assay to determine the activity at the organic phase” for methodology). The results demonstrated a protease inhibitory activity of 57.21 ± 0.009%. Subsequently, the fraction representing 99.08% purity was analyzed for compound identification, facilitating partial purification for further detailed analysis of GC-MS.

#### GC-MS analysis

**Fig. 10:**
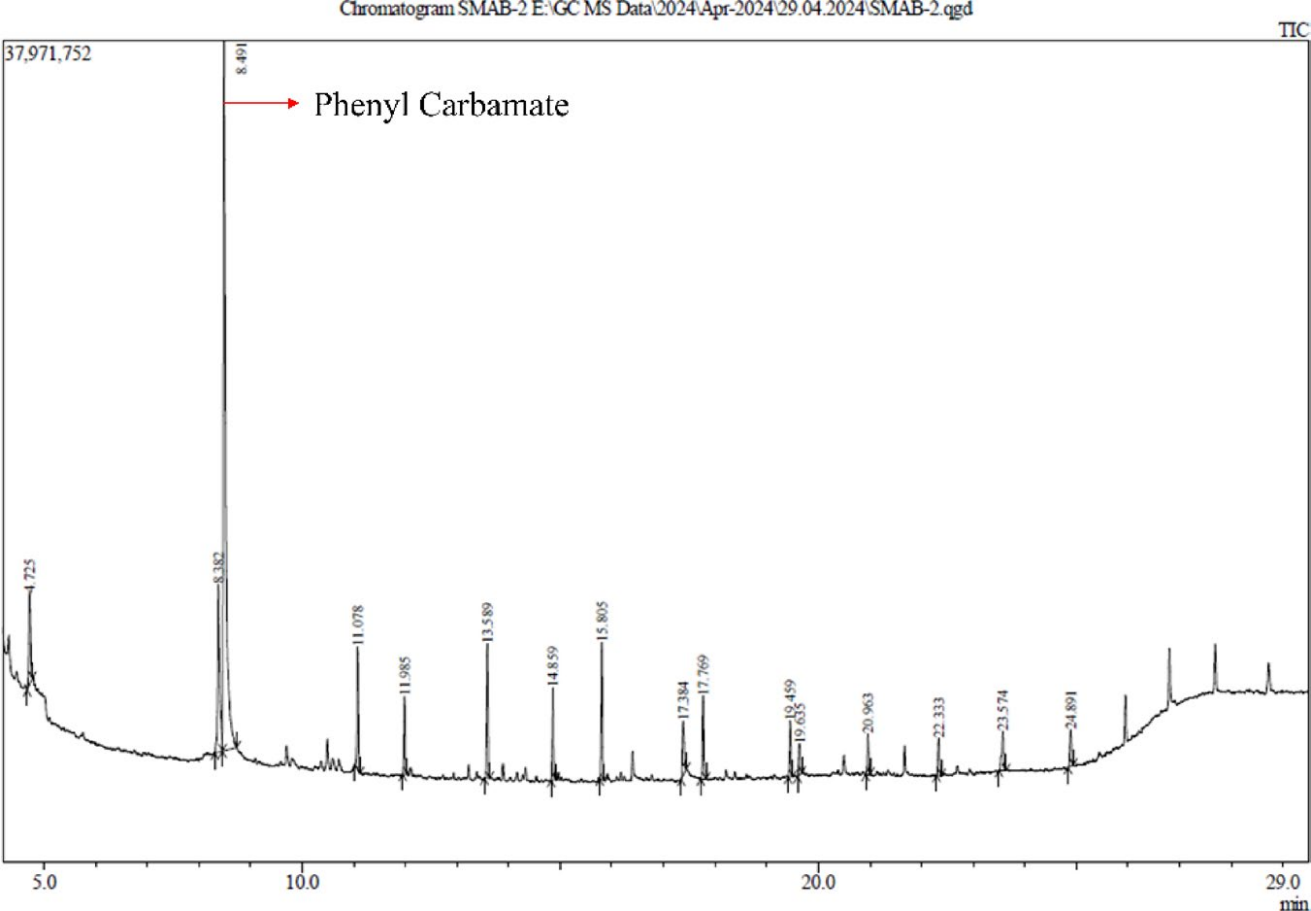
GC-MS chromatogram identifying the compounds present in the partially purified fraction from UPLC analysis of *Streptomyces globosus* VITSMAB2.

**Fig. 11:**
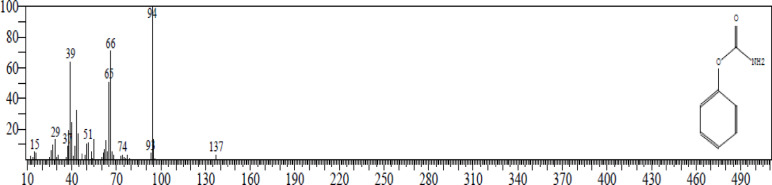
Chromatogram of the phenyl carbamate peak observed in GC-MS analysis.

GC-MS analysis is significant for its capability to accurately identify and quantify the chemical compounds in a sample. By examining the effective fractions from UPLC, GC-MS offers comprehensive details about the molecular structure and concentration of the compounds. The highest abundance compound found was phenyl carbamate (137.138 g/mol), with an area percentage of 47.23%. The GC-MS plots are represented in Figs. 10 and 11, and the results are documented in Table 4.

**Table 4:** GC-MS data of the identified compounds.

Peak	Retention time	Area %	Height%	Compound name
1	4.725	4.06	5.81	Cyclotrisiloxane, hexamethyl-
2	8.382	10.22	8.75	Cyclotetrasiloxane, octamethyl-
**3**	**8.491**	**47.23**	**37.13**	**Phenyl carbamate**
4	11.078	5.02	6.42	Cyclopentasiloxane, decamethyl-
5	11.985	3.04	4.06	3-Tetradecene, (Z)-
6	13.589	5.30	7.01	Cyclohexasiloxane, dodecamethyl-
7	14.859	3.36	4.84	3-Tetradecene
8	15.805	5.30	7.19	Hexasiloxane, 1,1,3,3,5,5,7,7,9,9,11,11-dodecamethyl-
9	17.384	2.71	2.83	1-Hexadecene
10	17.769	3.32	4.36	Cyclooctasiloxane, hexadecamethyl-
11	19.459	2.18	2.95	1,1,1,3,5,7,9,11,11,11-Decamethyl-5-(trimethyl siloxy)hexasiloxane
12	19.635	1.29	1.64	3-Eicosene, (E)-
13	20.963	1.62	2.12	1,1,1,3,5,7,9,11,11,11-Decamethyl-5-(trimethyl siloxy)hexasiloxane
14	22.333	1.53	1.95	Benzeneethanamine, N-[(pentafluorophenyl)methylene]-. beta.,3,4-tris[(trimethylsilyl)oxy]-
15	23.574	2.20	2.06	Octasiloxane, 1,1,3,3,5,5,7,7,9,9,11,11,13,13,15,15-hexadecamethyl-
16	24.891	1.63	1.89	Heptasiloxane, hexadecamethyl-

### Molecular docking analysis of the PI with SARS-CoV-2 m-Protease (cysteine protease)

**Fig. 12:**
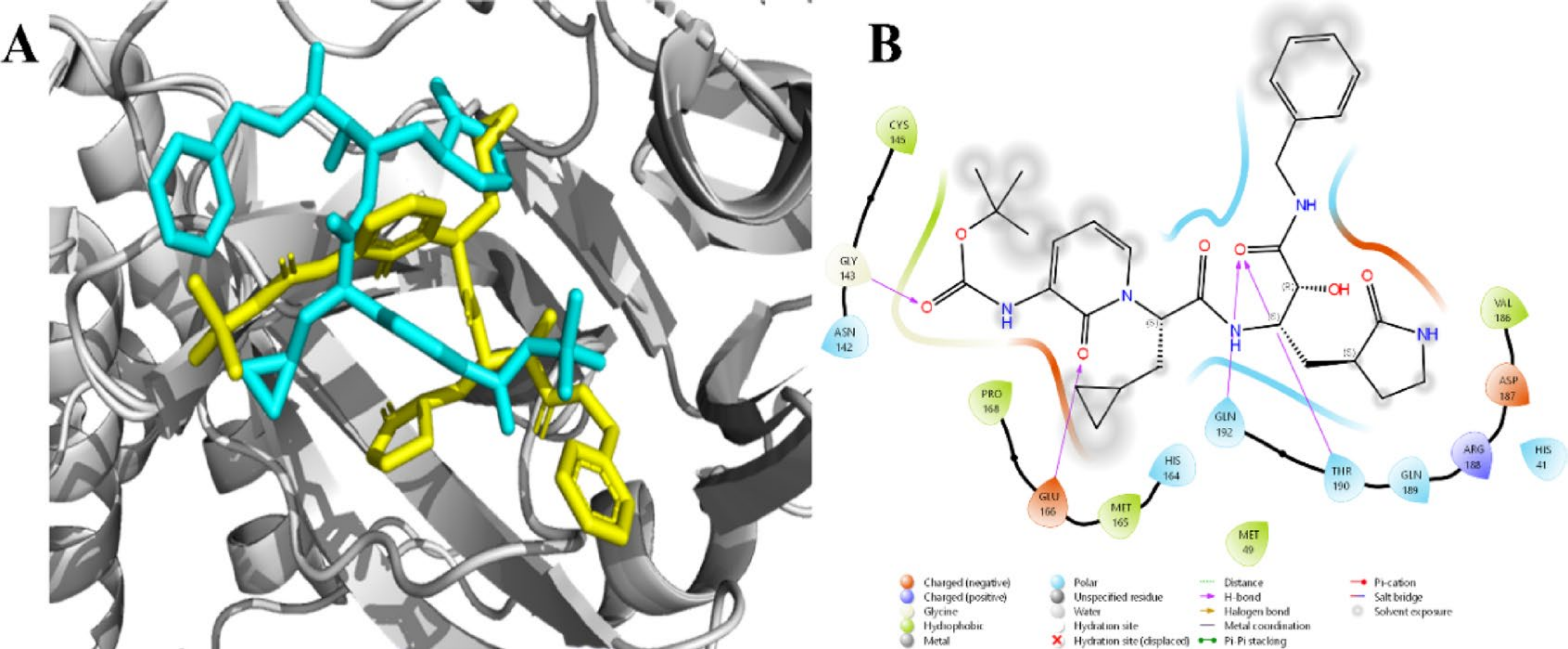
2-Dimensional representation illustrating the interacting residues of O6K with m-Pro (**A**) Redocked poses of the complex (**B**) Interacting amino acid residues.

**Fig. 13:**
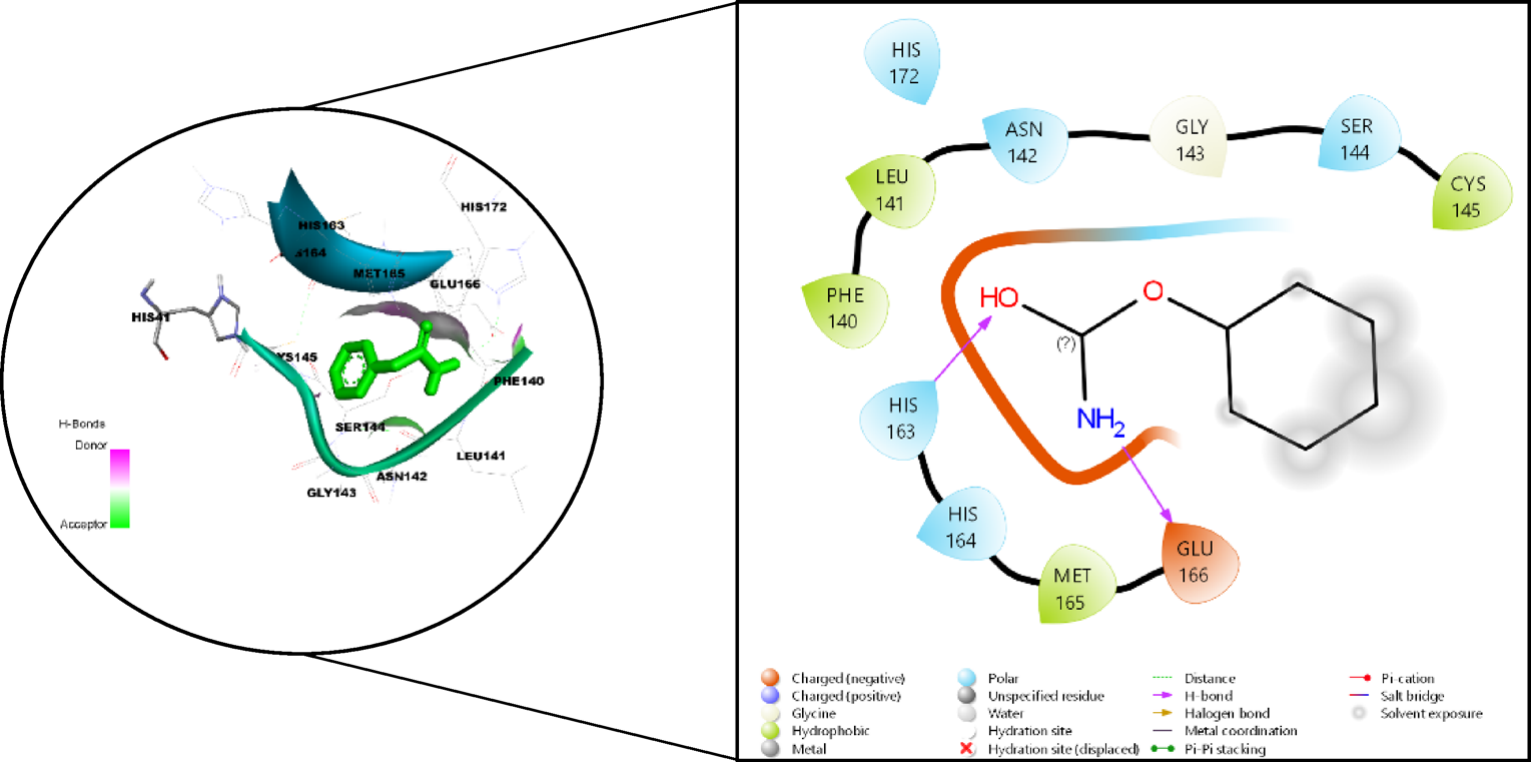
2-D representation illustrating the interacting residues of phenyl carbamate with m-Pro.

The data presented in Table 5 provides data on the binding energies, spatial parameters, and amino acid interactions of two ligands, cofactor (O6K) and phenyl carbamate, likely obtained from molecular docking simulations. The redocked structure shown in Fig. 12A illustrates the binding of 06 K at its active site, with a binding energy of − 7.5 kcal/mol. Hydrogen bond interactions are observed with GLY143, GLU166, THR190, and GLN192, while additional interactions involve HIS41, MET49, ASN142, CYS145, MET165, HIS164, PRO168, VAL186, ASP187, ARG188, and GLN189 (Fig. 12B). The grid box center for O6K and phenyl carbamate is located at X: 11.514, Y: − 1.286, Z: 21.214, with dimensions of X: 36, Y: 42, Z: 36. Conversely, phenyl carbamate shows binding energy of − 4.5 kcal/mol, with hydrogen bond interactions involving HIS163 and GLU166, and other interactions with PHE140, LEU141, ASN142, GLY143, SER144, CYS145, HIS164, MET165, and HIS172 (Fig. 13). Despite phenyl carbamate exhibiting lower binding energy compared to O6K, its inhibitory potential to occupy the same substrate-binding pocket and interact with catalytically essential residues highlights its inhibitory potential. The proximity of phenyl carbamate to CYS145, coupled with stabilizing interactions with neighbouring residues (HIS163, GLU166, and HIS164), may hinder the nucleophilic activity of the catalytic cysteine and disrupt the proteolytic processing of viral polyproteins. The consistent and uniform binding of phenyl carbamate at the same active site as O6K underscores its viability as a promising candidate for Mpro inhibition.

**Table 5:** Molecular Docking interactions of the cofactor and phenyl carbamate with M-pro.

Ligand	Binding Energy (kcal/mol)	H- bond interaction (Amino acid residues)	Other interaction (Amino acid residues)
Cofactor (O6K)	− 7.5	GLY143, GLU166, THR190, GLN192	HIS41, MET49, ASN142, CYS145, MET165, HIS164, PRO168, VAL186, ASP187, ARG188, GLN189
Phenyl carbamate	− 4.5	HIS163, GLU166	PHE140, LEU141, ASN142, GLY143, SER144, CYS145, HIS164, MET165, HIS172

### Drug-likeness, and ADME/T characteristics of the PI compound

The data presented in Table 6 presents detailed information on the phytochemical phenyl carbamate, including its PubChem ID, molecular weight (MW), Lipinski’s rule of five parameters (number of violations), and drug-likeness properties. Phenyl carbamate has a PubChem ID of 5,458,461 and a molecular weight of 137.14 g/mol. It shows zero violations of Lipinski’s rule of five, indicating compliance with drug-likeness guidelines. The molecule has one hydrogen bond acceptor (HBA), two hydrogen bond donors (HBD), and two rotatable bonds (NRB). Its topological polar surface area (TPSA) is 52.32 Å², and it has a LogP value of 1.84, suggesting favorable pharmacokinetic properties. Overall, this data provides insights into phenyl carbamate’s molecular characteristics and drug-likeness profile, aiding in its pharmaceutical evaluation and potential drug development processes.

**Table 6:** Drug-likeness properties of phenyl carbamate.

Compound	PUBCHEM ID	Lipinski’s rule of 5					
		MW^a^	NHA^b^	NHD^c^	NRB^d^	TPSA^e^	LogP
Phenyl carbamate	5,458,461	137.14	3	1	2	52.32	1.84

^a^ Molecular weight; ^b^ No. of H bond Acceptor; ^c^ No. of H bond Donor; ^d^ No. of Rotatable bonds; ^e^ Topological polar surface area.

**Fig. 14:**
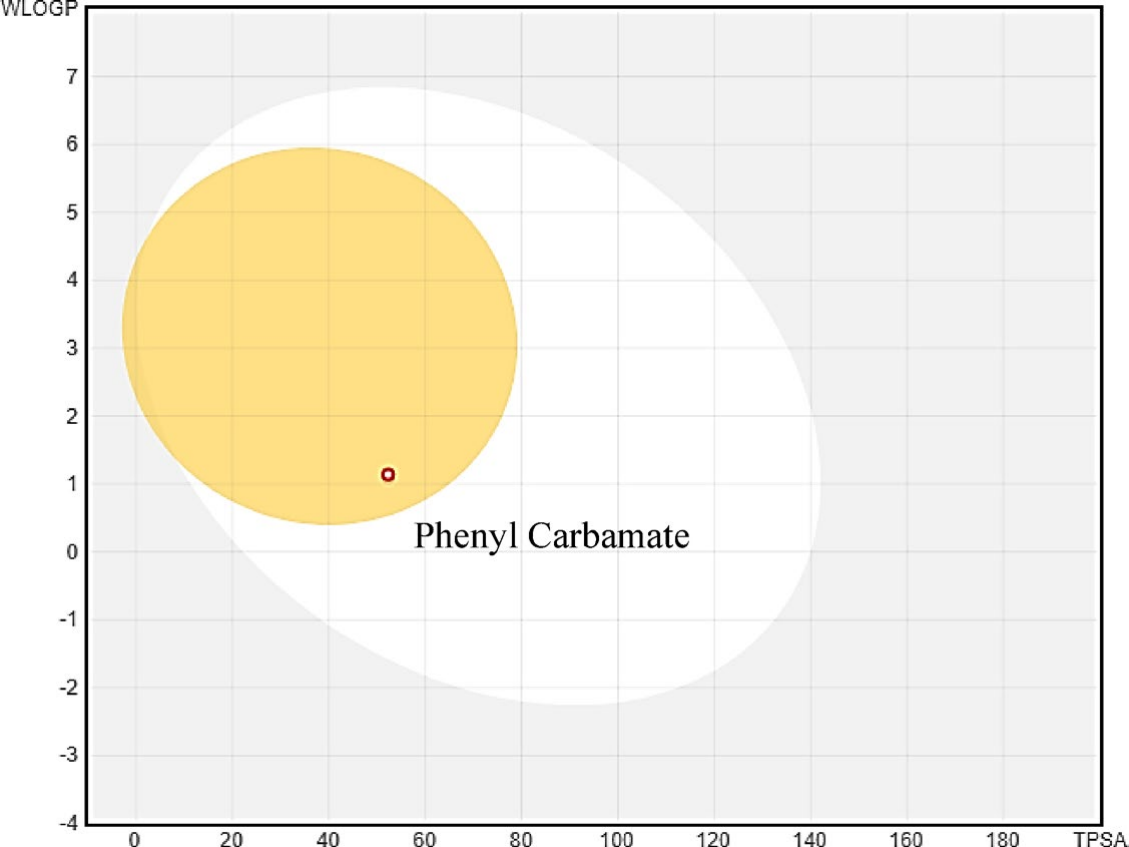
Boiled egg model representation of phenyl carbamate: The white region indicates the predicted GI absorption, while the yellow region denotes the predicted BBB permeability.

The data presented in Table 7 provides technical information regarding the ADMET profile of a compound. Specifically, it includes details on gastrointestinal (GI) absorption, water solubility, blood-brain barrier (BBB) permeability, CYP2D6 inhibition and substrate status, renal OCT2 substrate status, oral rat acute toxicity LD_50_, maximum tolerated dose in humans, and AMES toxicity. The compound exhibits high GI absorption, indicating efficient uptake in the GI tract, and is highly water-soluble, suggesting good dissolution properties. It permeates the blood-brain barrier, indicating potential central nervous system activity. It is neither a CYP2D6 inhibitor nor a substrate, suggesting minimal interaction with this cytochrome P450 enzyme. Additionally, it is not a renal OCT2 substrate, indicating it is not actively transported by this renal transporter. The oral rat acute toxicity LD_50_ is 2.105 mol/kg, indicating a moderate level of acute toxicity in rats. The maximum tolerated dose in humans is 1.102 log/mg/kg/day, providing insight into the compound’s safety profile for human dosing. Lastly, the compound does not exhibit AMES toxicity, suggesting it does not induce genetic mutations. An egg-boiled model^20^ (indicating GI absorption and BBB permeability) representation is also depicted in Fig. 14.

**Table 7:** Pharmacokinetics properties of phenyl carbamate.

Absorption	Distribution		Metabolism
GI absorption	Water solubility	BBB permeability	CYP2D6 onhibitor
High	Very Soluble	Yes	No

Excretion	Toxicity		
Renal OCT2 substrate	Oral rat acute toxicity LD50 (mol/kg)	Max tolerated dose (Human) (log/mg/kg/day)	AMES toxicity
No	2.105	1.102	No

### MD simulation of the pure compound with SARS-CoV-2 m-protease

**Fig. 15:**
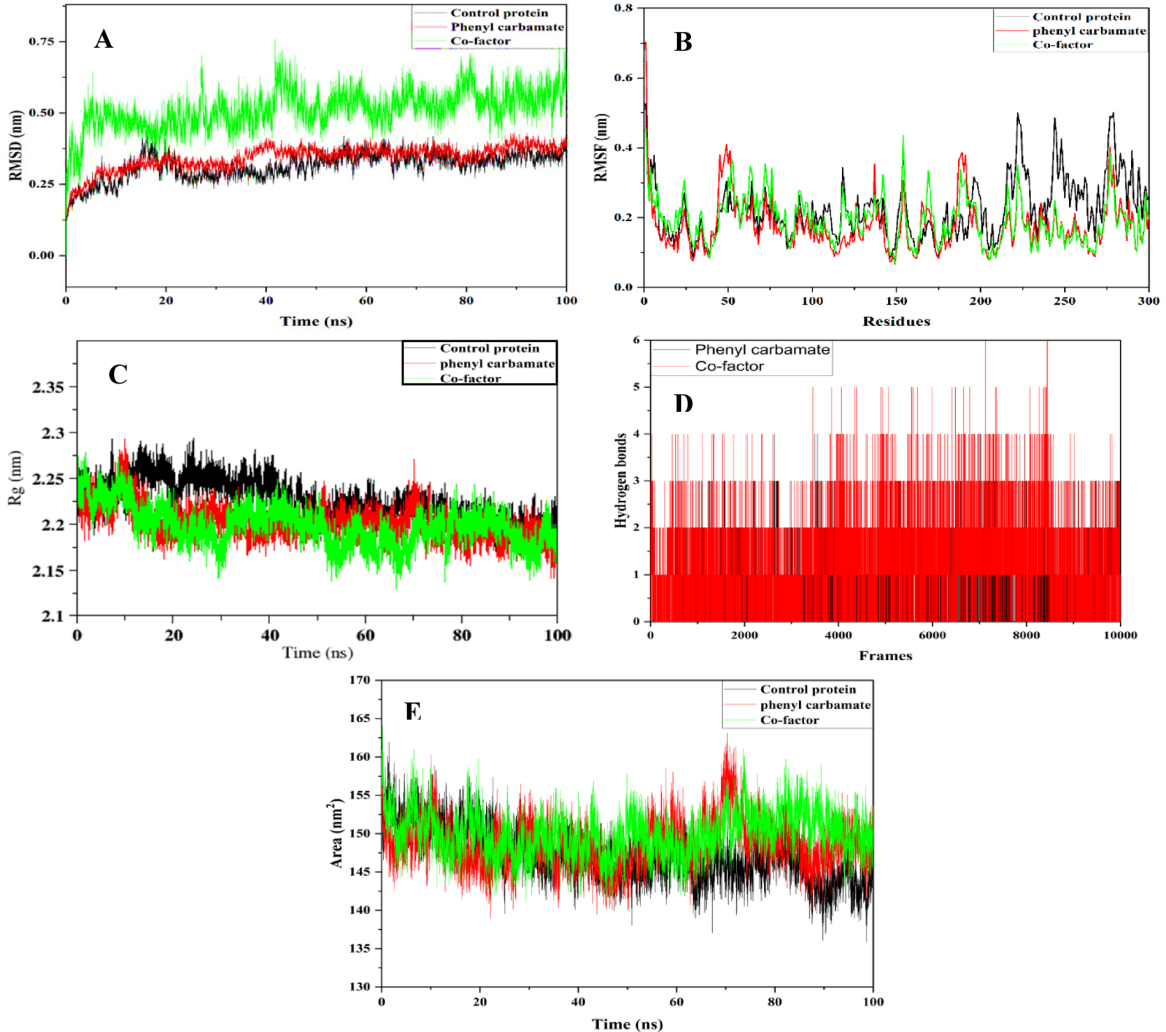
MDS analysis of 100ns run. (**A**) Root mean square deviation (RMSD) plot; (**B**) Root mean square fluctuations (RMSF) plot; (**C**) Radius of gyration (Rg) plot; (**D**) Hydrogen bonding plot; (**E**) Solvent accessible surface area (SASA) plot.

MD simulation of the complex was performed to evaluate the stability of the protein under physiological conditions. The unbound M-pro displayed an average RMSD of 0.315 nm, suggesting structural instability. In contrast, when bound with phenyl carbamate, M-pro demonstrates an average RMSD of 0.344 nm, suggesting a relatively stable conformation compared to O6K, which exhibits an average RMSD of 0.512 nm. Notably, during the initial 30 ns of simulation, M-pro displays high fluctuation, while in the subsequent stages (35–100 ns), it adopts a more stable conformation, as depicted in Fig. 15A. Hence, it can be inferred that phenyl carbamate assumes a rigid conformation by forming stable bonds with M-pro.

The RMSF analysis, consistent with RMSD findings, demonstrates a decreased deviation of amino acids from their native states. The unbound protein displays a higher amino acid fluctuation of 0.225 nm, contrasting with M-pro bound to phenyl carbamate, which exhibits a minimal fluctuation of 0.179 nm. Comparatively, M-pro bound with phenyl carbamate shows lesser deviations than the M-pro bound with O6K, with a fluctuation of 0.195 nm. This trend is depicted in Fig. 15B, indicating that the average fluctuations of amino acids around the active sites of M-pro bound to phenyl carbamate were 0.166 nm, indicating conformational stability in contrast to the unbound M-pro with average fluctuations of 0.191 nm and M-pro bound with O6K with average fluctuations of 0.189 nm around the active sites. These findings affirm the stability and rigidity of phenyl carbamate within the binding pocket throughout the simulation period.

The Rg serves as a valuable metric for assessing the shape and compactness of proteins, providing insights into the spatial arrangement of their constituent atoms. Our Rg analysis reveals average values of 2.22 nm and 2.19 nm for unbound M-pro and M-pro bound with O6K, respectively. Notably, an average Rg value of 2.20 nm is observed for M-pro bound with phenyl carbamate, elucidating the flexibility and rigidity of this interaction. As illustrated in Fig. 15C, a discernible decrease in the Rg value of M-pro bound with phenyl carbamate occurs between 20 and 50 ns, followed by a minor decline from 60 to 100 ns. This structural alteration is further supported by the RMSD plot of M-pro bound with phenyl carbamate, which exhibits stability from 50 to 100 ns.

Hydrogen bonding interactions between the protein and ligand are instrumental in assessing polar interactions. Our analysis reveals an average hydrogen bonding value of 1.20 for M-pro bound with O6K, contrasting with a lower value of 0.677 for M-pro bound with phenyl carbamate, as evidenced in Fig. 15D. These findings are corroborated by our docking analysis, which demonstrates a higher count of hydrogen bonds in the cofactor-bound M-pro compared to the phenyl carbamate-bound counterpart. This discrepancy underscores the presence of robust and stable interactions facilitated by O6K, likely supported by additional non-polar interactions such as van der Waals forces.

The unbound M-pro exhibited a SASA value of 147.20 nm², suggesting full exposure and availability for molecular interactions. Conversely, when M-pro was bound with phenyl carbamate, the average SASA value decreased to 145.69 nm², as depicted in Fig. 15E. In contrast, the bound O6K exhibited a higher average SASA value of 150.006 nm², indicating greater solvent accessibility. This implies that the binding of phenyl carbamate to M-pro restricts its accessibility, potentially hindering the usual functions of M-pro.

The MD simulation results demonstrate that phenyl carbamate confers superior stability to M-pro compared to O6K. The RMSD analysis shows lower structural deviations, while RMSF indicates reduced residue flexibility around the active site. The Rg values reveal consistent compactness throughout the simulation. Despite fewer hydrogen bonds, the complex remains stable due to strong hydrophobic interactions. Furthermore, the lower SASA value confirms reduced solvent exposure, validating the enhanced rigidity and stability of the M-pro–phenyl carbamate complex.

## Discussion

The present study reports the isolation of a protease-inhibiting actinobacterium from a high-altitude soil sample and the identification of its major bioactive metabolite, phenyl carbamate. The isolate, *Streptomyces globosus* VITSMAB2, was obtained using AIA and SCA media and characterized through morphological and biochemical analyses. Protease inhibition assays confirmed its strong inhibitory potential, primarily against cysteine proteases (papain) and subsequently against serine proteases (trypsin). Chromatographic and spectrometric analyses (UPLC and GC-MS) identified phenyl carbamate as the dominant compound contributing to this activity. Pharmacokinetic predictions indicated favorable drug-like properties, including high gastrointestinal absorption, water solubility, and blood–brain barrier permeability, supporting its suitability as a potential therapeutic candidate.

To explore its antiviral potential, molecular docking and molecular dynamics (MD) simulations were performed against the SARS-CoV-2 main protease (Mpro). Phenyl carbamate exhibited a binding energy of − 4.5 kcal/mol compared to − 7.5 kcal/mol for the co-crystallized ligand O6K, forming hydrogen bonds with HIS163 and GLU166 and hydrophobic interactions with key catalytic residues such as CYS145, HIS164, and HIS172. The 100 ns MD simulation demonstrated the complex’s stability (average RMSD 0.344 nm) with reduced amino acid fluctuations (RMSF 0.179 nm) and consistent compactness (Rg 2.20 nm), suggesting a stable and functionally relevant binding conformation. These results collectively indicate that phenyl carbamate occupies the same substrate-binding pocket as O6K, interacting with the catalytically crucial CYS145 residue of the CYS145–HIS41 dyad. This correlation between enzyme mechanism and docking data highlights phenyl carbamate’s reliability as a potential Mpro inhibitor. Notably, no previous studies have reported antiviral or protease-inhibitory activity of phenyl carbamate, positioning this work as a pioneering exploration and a valuable starting point for future in vitro and in vivo investigations.

In the broader context, extensive research has been devoted to discovering SARS-CoV-2 Mpro inhibitors through both in silico and experimental approaches. Qiao et al. (2021) designed 32 inhibitors based on boceprevir and telaprevir, six of which protected cells from infection, while two showed antiviral efficacy in a transgenic mouse model^21^. Jin et al. (2020) identified the mechanism-based inhibitor N3 and, through virtual and high-throughput screening of over 10,000 compounds, reported six Mpro inhibitors with IC₅₀ values between 0.67 and 21.4 µM, with Ebselen exhibiting notable antiviral activity^22^. Kumar et al. (2025) used pharmacophore modeling and large-scale virtual screening of 200 million compounds to identify two potent Mpro inhibitors, later expanding their research (2025) to report fluoro-deoxy-glucose folate (FDGF) and N-(2-fluoro-3-(6-O-glucosylpropyl-azomycin)) as dual inhibitors of Mpro and spike protein, with strong binding affinities (− 8.6 to − 9.9 kcal/mol)^23^. Khan et al. (2025) synthesized quinoline-4-carboxamide derivatives (2e, 6b, 6c) as dual BACE-1 and Mpro inhibitors, with compound 6c showing potent binding and in vivo safety^24^. Soulère et al. (2021) identified electrophilic compounds and repurposed drugs targeting the catalytic CYS145 residue^25^, while Ibrahim et al. (2021) demonstrated that erylosides B from Red-Sea terpenes exhibited superior Mpro binding (Δ Gbinding − 51.9 kcal/mol) compared to lopinavir (− 33.6 kcal/mol)^26^. Collectively, these studies highlight the global effort to develop structurally diverse and mechanistically validated Mpro inhibitors.

By comparison, the current study provides both biochemical and computational evidence that a naturally derived compound, phenyl carbamate, effectively targets the catalytic CYS145 residue in SARS-CoV-2 Mpro. Its stable binding, confirmed through molecular docking and MD simulations, supports its candidacy as a novel inhibitor scaffold derived from actinomycetes. This finding bridges natural product discovery and targeted antiviral design, contributing new molecular insights to Mpro inhibition research.

Even in the post-pandemic era, this study maintains strong relevance. Continued investigation into Mpro inhibitors such as phenyl carbamate is essential for pandemic preparedness and the development of broad-spectrum antivirals targeting conserved viral proteases. Understanding the molecular interactions and stability of potential inhibitors ensures readiness against future coronavirus outbreaks and other emerging viral pathogens. Therefore, this work not only advances the discovery of natural metabolite-based antiviral agents but also contributes meaningfully to global health resilience and future therapeutic innovation.

## Conclusion

This study highlights the discovery of *Streptomyces globosus* VITSMAB2, a unique actinomycete isolated from a high-altitude environment, exhibiting remarkable protease inhibitory potential. The isolate produced phenyl carbamate as its dominant bioactive metabolite, which demonstrated strong inhibition of both cysteine and serine proteases. Analytical characterization confirmed phenyl carbamate as a chemically stable and pharmacologically promising compound with favorable drug-likeness and and provide preliminary predictions, including its ADME/T characteristics, including high gastrointestinal absorption and blood–brain barrier permeability. Computational analyses revealed that phenyl carbamate binds effectively to the catalytic pocket of the SARS-CoV-2 main protease, interacting with key residues critical for viral replication. Molecular dynamics simulations further confirmed the stability and compactness of the phenyl carbamate–Mpro complex, suggesting a robust and functionally relevant interaction. These findings collectively propose phenyl carbamate as a potential natural inhibitor with significant antiviral implications. Overall, this work establishes a strong foundation for exploring actinomycete-derived metabolites as novel therapeutic agents. By integrating microbiological, biochemical, and computational insights, the study underscores the potential of natural products in antiviral drug discovery. Phenyl carbamate thus represents not only a promising lead compound but also a valuable step forward in developing sustainable, nature-inspired strategies for combating viral infections.

## Future prospects

Phenyl carbamate demonstrates promising pharmacokinetic characteristics, making it a strong candidate for therapeutic development against viral infections, including SARS-CoV-2. Although certain challenges, such as the need for molecular refinement, persist, advancements in cheminformatics, nanoformulation techniques, and structural modifications provide effective strategies to enhance its therapeutic potential. Phenyl carbamate may also serve as an adjuvant, complementing existing antiviral therapies to improve overall efficacy. Further investigation is necessary to confirm its in vivo effectiveness, evaluate its safety profile, and determine its clinical applicability. Comprehensive studies including viral inhibition assays, cytotoxicity evaluations, and in vivo trials are crucial to validate the compound’s therapeutic promise. This work establishes an early framework for exploring actinomycete-derived natural antivirals and emphasizes the value of natural products in the pursuit of new pharmaceutical agents. 

## Supplementary Information

Below is the link to the electronic supplementary material.

Supplementary Material 1

Supplementary Material 2

## Data Availability

All data generated or analyzed during this study are included in this article and its supplementary information files.
