# Supplementary material for: Computational insights into a protease inhibitor from Streptomyces globosus VITSMAB-2 molecular docking and dynamics simulations against SARS-CoV-2 main protease
Source: Sci Rep. 2025 Dec 13;16:1778. doi: 10.1038/s41598-025-31329-y (PMC12804924; doi:10.1038/s41598-025-31329-y)
Supplement: Supplementary file 1 — Supplementary Material 1 [file 41598_2025_31329_MOESM1_ESM.docx]

**Computational Insights into a Protease Inhibitor from *Streptomyces globosus* VITSMAB-2 Molecular Docking and Dynamics Simulations against SARS-CoV-2 Main Protease**

**Shatakshi Mishra^a#^, Stany Bala Kumar^a#^, Aparana Kumari^b^, K.V. Bhaskara Rao^b^***

**^a^** School of Bio Sciences and Technology, Vellore Institute of Technology, Vellore, 632014, Tamil Nadu, India

**^b*^** Marine Biotechnology Laboratory, Department of Biomedical Sciences, School of Bio Sciences and Technology, Vellore Institute of Technology, Vellore, 632014, Tamil Nadu, India

# Equal Contributions

**SUPPLEMENTARY DATA**

GenBank ID: PP809233.1

<https://www.ncbi.nlm.nih.gov/nuccore/2733407846?log$=activity>


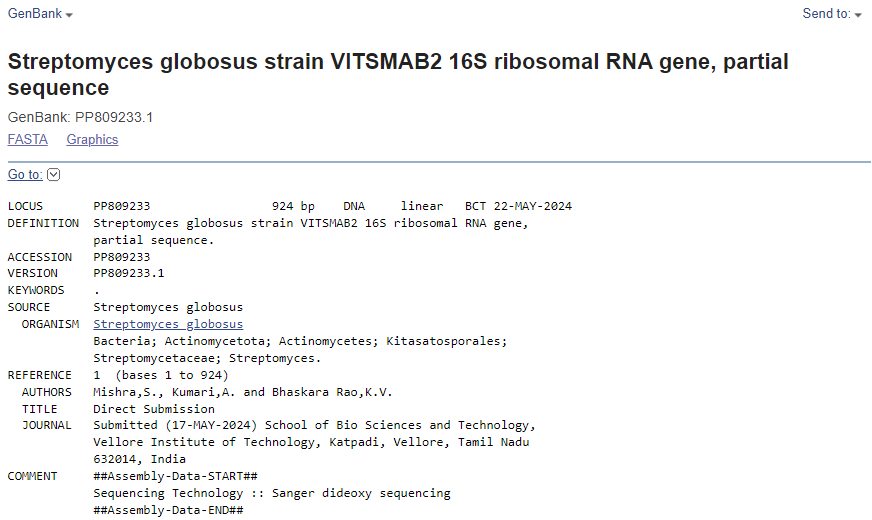


**Supplementary Table 1** Quantitative determination of trypsin Inhibition by SMAB2.

| **SAMPLE** | **ABSORBANCE AT 410 nm** | | | **AVERAGE± S. D** | **PERCENTAGE (%)** |
| --- | --- | --- | --- | --- | --- |
| CONTROL | 0.446 | 0.441 | 0.45 | 0.445± 0.004 | 0 |
| **SMAB2** | **0.183** | **0.185** | **0.179** | **0.182± 0.003** | **59.08** |
| SMAB6 | 0.201 | 0.202 | 0.205 | 0.202± 0.002 | 54.52 |
| SMAB15 | 0.197 | 0.19 | 0.193 | 0.193± 0.003 | 56.61 |
| SMAB16 | 0.19 | 0.193 | 0.191 | 0.191± 0.001 | 57.06 |
| SMAB17 | 0.346 | 0.351 | 0.348 | 0.348± 0.002 | 21.83 |
| SMAB18 | 0.311 | 0.315 | 0.309 | 0.311± 0.003 | 30.06 |
| SMAB19 | 0.401 | 0.402 | 0.403 | 0.402± 0.001 | 9.79 |

**Supplementary Table 2** Quantitative Determination of Papain Inhibition by SMAB2.

| **SAMPLE** | **ABSORBANCE AT 410 nm** | | | **AVERAGE± S.D** | **PERCENTAGE (%)** |
| --- | --- | --- | --- | --- | --- |
| CONTROL | 0.381 | 0.385 | 0.383 | 0.383± 0.002 | 0 |
| **SMAB2** | **0.143** | **0.144** | **0.143** | **0.143± 0.0005** | **62.57** |
| SMAB6 | 0.201 | 0.202 | 0.202 | 0.201± 0.0005 | 47.34 |
| SMAB15 | 0.149 | 0.15 | 0.15 | 0.149± 0.0005 | 60.92 |
| SMAB16 | 0.171 | 0.173 | 0.173 | 0.172± 0.0011 | 55.004 |
| SMAB17 | 0.246 | 0.247 | 0.246 | 0.246± 0.0005 | 35.68 |
| SMAB18 | 0.319 | 0.318 | 0.32 | 0.319± 0.001 | 16.71 |
| SMAB19 | 0.244 | 0.24 | 0.243 | 0.242± 0.002 | 36.72 |

**Supplementary Table 3** Quantitative assay to determine the activity at the organic phase.

| SAMPLE | ABSORBANCE AT 410 nm | | | AVERAGE± S. D | PERCENTAGE (%) |
| --- | --- | --- | --- | --- | --- |
| CONTROL | 0.381 | 0.385 | 0.379 | 0.381± 0.003 | 0 |
| SMAB 2 (Organic phase) | 0.143 | 0.15 | 0.159 | 0.150± 0.008 | 60.29% |
| SMAB 2 (Aqueous phase) | 0.28 | 0.28 | 0.273 | 0.277± 0.004 | 27.29% |
