# Supplementary material for: Computational insights into a protease inhibitor from Streptomyces globosus VITSMAB-2 molecular docking and dynamics simulations against SARS-CoV-2 main protease
Source: Sci Rep. 2025 Dec 13;16:1778. doi: 10.1038/s41598-025-31329-y (PMC12804924; doi:10.1038/s41598-025-31329-y)
Supplement: Supplementary file 2 — Supplementary Material 2 [file 41598_2025_31329_MOESM2_ESM.pdf]

Computational Insights into a Protease Inhibitor from *Streptomyces globosus* VITSMAB-2 Molecular Docking and Dynamics Simulations against SARS-CoV-2 Main Protease

Shatakshi Mishra[a#], Stany Bala Kumar[a#], Aparana Kumari[b], K.V. Bhaskara Rao[b*]

[a] School of Bio Sciences and Technology, Vellore Institute of Technology, Vellore, 632014, Tamil Nadu, India

[b*] Marine Biotechnology Laboratory, Department of Biomedical Sciences, School of Bio Sciences and Technology, Vellore Institute of Technology, Vellore, 632014, Tamil Nadu, India

# Equal Contributions

SUPPLEMENTARY DATA

GenBank ID: PP809233.1

https://www.ncbi.nlm.nih.gov/nuccore/2733407846?log$=activity

GenBank ▾ Send to: ▾

Streptomyces globosus strain VITSMAB2 16S ribosomal RNA gene, partial sequence

GenBank: PP809233.1

FASTA Graphics

Go to:

```
LOCUS       PP809233                 924 bp    DNA     linear   BCT 22-MAY-2024
DEFINITION  Streptomyces globosus strain VITSMAB2 16S ribosomal RNA gene,
            partial sequence.
ACCESSION   PP809233
VERSION     PP809233.1
KEYWORDS    .
SOURCE      Streptomyces globosus
  ORGANISM  Streptomyces globosus
            Bacteria; Actinomycetota; Actinomycetes; Kitasatosporales;
            Streptomycetaceae; Streptomyces.
REFERENCE   1  (bases 1 to 924)
  AUTHORS   Mishra,S., Kumari,A. and Bhaskara Rao,K.V.
  TITLE     Direct Submission
  JOURNAL   Submitted (17-MAY-2024) School of Bio Sciences and Technology,
            Vellore Institute of Technology, Katpadi, Vellore, Tamil Nadu
            632014, India
COMMENT     ##Assembly-Data-START##
            Sequencing Technology :: Sanger dideoxy sequencing
            ##Assembly-Data-END##
```

Supplementary Table 1 Quantitative determination of trypsin Inhibition by SMAB2.

**SAMPLE**	**ABSORBANCE AT 410 nm**			**AVERAGE± S. D**	**PERCENTAGE (%)**
CONTROL	0.446	0.441	0.45	0.445± 0.004	0
**SMAB2**	**0.183**	**0.185**	**0.179**	**0.182± 0.003**	**59.08**
SMAB6	0.201	0.202	0.205	0.202± 0.002	54.52
SMAB15	0.197	0.19	0.193	0.193± 0.003	56.61
SMAB16	0.19	0.193	0.191	0.191± 0.001	57.06
SMAB17	0.346	0.351	0.348	0.348± 0.002	21.83
SMAB18	0.311	0.315	0.309	0.311± 0.003	30.06
SMAB19	0.401	0.402	0.403	0.402± 0.001	9.79

Supplementary Table 2 Quantitative Determination of Papain Inhibition by SMAB2.

**SAMPLE**	**ABSORBANCE AT 410 nm**			**AVERAGE± S.D**	**PERCENTAGE (%)**
CONTROL	0.381	0.385	0.383	0.383± 0.002	0
**SMAB2**	**0.143**	**0.144**	**0.143**	**0.143± 0.0005**	**62.57**
SMAB6	0.201	0.202	0.202	0.201± 0.0005	47.34
SMAB15	0.149	0.15	0.15	0.149± 0.0005	60.92
SMAB16	0.171	0.173	0.173	0.172± 0.0011	55.004
SMAB17	0.246	0.247	0.246	0.246± 0.0005	35.68
SMAB18	0.319	0.318	0.32	0.319± 0.001	16.71
SMAB19	0.244	0.24	0.243	0.242± 0.002	36.72

Supplementary Table 3 Quantitative assay to determine the activity at the organic phase.

SAMPLE	ABSORBANCE AT 410 nm			AVERAGE± S. D	PERCENTAGE (%)
CONTROL	0.381	0.385	0.379	0.381± 0.003	0
SMAB 2 (Organic phase)	0.143	0.15	0.159	0.150± 0.008	60.29%
SMAB 2 (Aqueous phase)	0.28	0.28	0.273	0.277± 0.004	27.29%

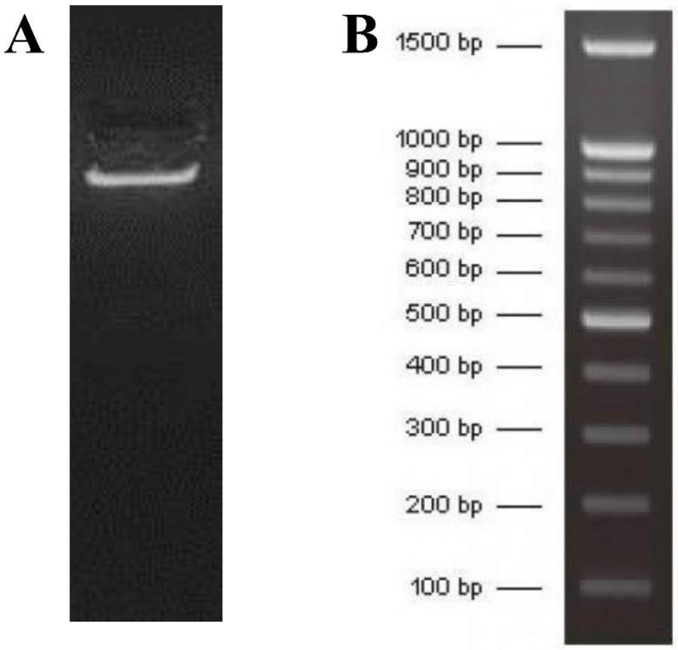

Supplementary Fig. 1 (A) PCR gel image of SMAB2 demonstrating the successful amplification of target DNA **(B)** PCR ladder
